# The Relationship Between Early Adversity, Cardiovascular Risk, and Cognitive Health

**DOI:** 10.1002/alz70860_103584

**Published:** 2025-12-23

**Authors:** Liang Wang

**Affiliations:** ^1^ Dementias Platform UK, OXFORD, Oxfordshire, United Kingdom; University of Oxford, OXFORD, Oxfordshire, United Kingdom

## Abstract

**Background:**

Early adversity (EA) refers to low socioeconomic status (SES) and emotional abuse, is associated with an increased risk of cardiovascular risk factors such as heart disease, hypertension, and hyperlipidemia. These cardiovascular factors, in turn, may contribute to cognitive decline, impairment, and dementia in later life. This study investigates the intricate relationships between EA, cardiovascular risk factors, and cognitive health, focusing on the potential mediating role of cardiovascular mediating role of cardiovascular factors. By synthesising existing evidence, we identify key pathways that may contribute to cognitive decline and highlight directions for future investigation and intervention.

**Method:**

A comprehensive literature search was conducted following PRISMA guidelines in databases including Medline, Embase, PsycINFO, and Global Health, covering publications from January 2000 to February 2024. Targeted keywords such as “early adversity,” “cardiovascular risk,” and “cognitive health” guided study selection. Risks of bias were assessed using the Quality In Prognosis Studies (QUIPS) tool. Building on the findings of this review, future research will involve cohort data analysis (e.g., UK Biobank, ELSA) to further examine the mediating role of cardiovascular risk in the EA‐cognitive health relationship. Planned analyses include regression modelling and mediation analysis to assess these relationships quantitatively.

**Result:**

Nine studies were included in the final analysis from 2008 to 2024 across high‐income and upper‐middle‐income countries. The findings showed mixed evidence on the relationship between EA, cardiovascular risk, and cognitive health. While some studies identified significant associations, others found no clear links. The role of cardiovascular risk as a mediator between EA and cognitive health was also inconsistent. Cardiometabolic dysregulation (CMD) showed a weak but significant mediation effect in some studies, whereas hypertension was not supported as a mediator. These results underscore the complexity of these associations and highlight the need for further research across diverse populations.

**Conclusion:**

This study highlights the critical role of cardiovascular health in mitigating the long‐term cognitive effects of early adversity. While the systematic review identifies key gaps and potential mediating pathways, future data analysis will provide quantitative insights, enabling a more robust understanding of these relationships. These findings could inform targeted interventions to reduce the burden of cognitive disorders in vulnerable populations.

